# A Common SMAD7 Variant Is Associated with Risk of Colorectal Cancer: Evidence from a Case-Control Study and a Meta-Analysis

**DOI:** 10.1371/journal.pone.0033318

**Published:** 2012-03-21

**Authors:** Qibin Song, Beibei Zhu, Weiguo Hu, Liming Cheng, Hongyun Gong, Bin Xu, Xiawen Zheng, Li Zou, Rong Zhong, Shengyu Duan, Wei Chen, Rui Rui, Jing Wu, Xiaoping Miao

**Affiliations:** 1 Department of Oncology, Renmin Hospital of Wuhan University, Wuhan, China; 2 Department of Epidemiology and Biostatistics and MOE Key Lab of Environment and Health, School of Public Health, Tongji Medical College, Huazhong University of Science and Technology, Wuhan, China; 3 Department of Laboratory Medicine, Tongji Hospital, Tongji Medical College, Huazhong University of Science and Technology, Wuhan, China; IFOM, Fondazione Istituto FIRC di Oncologia Molecolare, Italy

## Abstract

**Background:**

A common genetic variant, rs4939827, located in *SMAD7*, was identified by two recent genome-wide association (GWA) studies to be strongly associated with the risk of colorectal cancer (CRC). However, the following replication studies yielded conflicting results.

**Method and Findings:**

We conducted a case-control study of 641 cases and 1037 controls in a Chinese population and then performed a meta-analysis, integrating our and published data of 34313 cases and 33251 controls, to clarify the relationship between rs4939827 and CRC risk. In our case-control study, the dominant model was significant associated with increased CRC risk [Odds Ratio (OR) = 1.46; 95% confidence interval (95% CI), 1.19–1.80]. The following meta-analysis further confirmed this significant association for all genetic models but with significant between-study heterogeneity (all *P* for heterogeneity <0.1). By stratified analysis, we revealed that ethnicity, sample size, and tumor sites might constitute the source of heterogeneity. The cumulative analysis suggested that evident tendency to significant association was seen with adding study samples over time; whilst, sensitive analysis showed results before and after removal of each study were similar, indicating the highly stability of the current results.

**Conclusion:**

Results from our case-control study and the meta-analysis collectively confirmed the significant association of the variant rs4939827 with increased risk of colorectal cancer. Nevertheless, fine-mapping of the susceptibility loci defined by rs4939287 should be imposed to reveal causal variant.

## Introduction

Colorectal cancer (CRC) is the third most common cancer and the fourth leading cause of cancer mortality worldwide [1]. Among the risk factors and causes for CRC, genetic component has strongly contributed to CRC development, which accounts for approximately 35% of total cases as reflected by twin- and family-based studies [2]. However, so far genetic factors have incompletely been characterized.

Genome-wide association (GWA) study has greatly contributed to identification of common genetic variants associated to common disease without prior knowledge of gene function. Several resent GWA studies have reported multiple novel susceptibility loci to colorectal cancer [3]–[11]._ENREF_7 Among these loci, the single nucleotide polymorphism (SNP, rs4939827), located in 18q21, has been strongly associated with risk of CRC by multiple GWA studies [3], [10]. Broderick et al. firstly identified rs4939827 in a GWA set of 620 cases and 960 controls and 3 replication sets of 7377 cases and 5867controls [10], and then Tenesa et al. further refined this finding in another comprehensive, phased-based GWA study comprising 16759 cases and 15545 controls [3]. Interestingly, rs4939827 maps to Mothers against decapentaplegic homolog 7 (*SMAD7*), a strong candidate gene in the famous transformation growth factor-β (TGF-β) pathway. SMAD7 acts as an intracellular antagonist of TGF-β signaling by recruiting SMURF to receptors for inactivation. Perturbation of SMAD7 and suppression of TGF-β signaling has been documented to involve in CRC [12]. Much attention has been drawn to this SNP; however, several follow-up studies cannot replicate the association [13]–[16], which may be due to the sample size. For instance, in Chinese population, Xiong et al. reported a significant association of this SNP with CRC risk [17], whereas Li et al. failed to replicate this association [13]. Similar controversial results were also seen in the replication studies in European [15], [16]. These results emphasize a need of additional replication for large sample size. Herein, we performed a replication study comprising 641cases and 1037 controls in a Chinese population. Moreover, meta-analysis is a method combing data together to make sample size exponential growth to get enough power to clarify inconsistent results in genetic association studies [18]. We further conducted a meta-analysis, combining current and previously published studies about rs4939827, to clarify the real relationship between this SNP and CRC risk.

## Materials and Methods

### Study populations

In this study, a total of 641 new CRC cases and 1037 cancer-free controls were enrolled from between 2009 and 2011 from Tongji Hospital of Huazhong University of Science and Technology, Wuhan, China. Cases had been histopathologically confirmed with primary colorectal cancer and had not received any treatment prior to blood samples collection. Controls were randomly selected from a subject pool of individuals who participate in health check-up programs at the same hospital in the same time period as the patients were enrolled. Controls were frequency matched to patients by age (±5 years) and gender. All subjects were unrelated ethnic Han Chinese living in Wuhan region. At recruitment, a 5-ml peripheral venous blood sample was collected from each subject after written informed consent was obtained. This study was approved by ethnics committee of Tongji Hospital of Huazhong University of Science and Technology.

### DNA isolation and genotyping

Genomic DNA was extracted from 5-mL of peripheral blood sample using the RelaxGene Blood System DP319-02 (Tiangen, Beijing, China) according to the manufacturer's instructions. The genotypes of rs4939827 were determined by using the TaqMan SNP Genotyping Assay (Applied Biosystems, Foster city, CA) on a 7900HT Fast Real-Time PCR System (Applied Biosystems, Foster city, CA). For quality control, 5%duplicated samples were randomly selected for to assess the reproducibility, with a concordance rate of 100%

### Statistical analysis

Pearson *χ^2^* test, fisher exact test, and *t* test were employed to evaluate the differences in distribution of demographic characteristics and genotypes between case and control groups, where appropriate. Goodness-of-fit χ^2^ test was adopted to assess Hardy-Weinberg Equilibrium (HWE) in the controls. Unconditional multivariate logistic regression analysis was used to estimate odds ratios (ORs) and their 95% confidence intervals (CIs) for the effect of rs4939827 genotypes on CRC risk, after adjusting for age and sex. To avoid the assumptions of genetic models, additive and dominant models for rs4939827 were also assessed. All statistical analyses were performed with the SPSS 12.0 software. A value of *P*<0.05 was considered representative of statistical significance.

### Meta-analysis of rs4939827 in association with CRC risk

To further investigate the association between rs4939827 and CRC risk, a meta-analysis based on the published studies was carried out according to the guidelines of Preferred Reporting Items for Systemic Reviews and Meta-Analyses statement (PRISMA) [19]. Systematic literature search updated to September, 2011 were performed in the PubMed and EMBase databases(Figure S1), using the search strategy based on combinations of the keywords “rs4939827 or 18q21” and “colorectal cancer, colorectal neoplasia or colorectal adenoma” without language restriction. References listed in the retrieved articles were also scanned. Reviews, comments, and letters were also checked for additional studies. Studies were included if they met the all of the following criteria: (a) assessment of the association between rs4939827 and CRC risk; (b) use of a case-control study or nested case-control study design; (c) information provided on genotype or allele frequency for risk estimates; (d) the genotype of controls is in Hardy-Weinberg equilibrium; (e) studies of humans. If the studies had overlapping subjects, only the study contained the largest population was finally included. Three reports were excluded due to lack of sufficient data for calculation of ORs after contacting with individual authors by E-mail [20]–[22].

The following data were extracted by two independent authors (B. Zhu & Q. Song): first author's last name, country of origin, publication year, predominant ethnicity of participants, sample size, study method and design, source of control groups (population- or hospital-based controls), genotyping method. Counts of alleles and genotypes in cases and controls were extracted or calculated from published data. Pooled frequency of the T allele in various ethnic populations was estimated using the inverse variance method previously described by Thakkinstian et al [23]. ORs and their 95% CIs as the metrics of effect size were re-calculated for the genotypes TT versus CC and CT versus CC. A dominant model was assumed for rs4939827, and an additive “per-allele” model and a recessive model were also considered. In this study, we used the Cochran's *Q* statistic to assess heterogeneity (heterogeneity was considered significant at *P*<0.1) [24]. The *I^2^* metric was applied to quantify heterogeneity irrespective of numbers of studies (*I^2^* = 0–25%, no heterogeneity; *I^2^* = 25–50%, moderate heterogeneity; *I^2^* = 50–75%, large heterogeneity; *I^2^* = 75–100%, extreme heterogeneity) [25]. A fixed-effects model, using Mantel-Haenszel method [26], was applied to pool data from studies when heterogeneity was negligible; otherwise, a random-effects model, using DerSimonian and Laird method, was applied [27]. Stratified analyses were performed, if feasible, according to ethnicity (European, Asian and mixed population), sample size (≤1000 and >1000 subjects), study design (GWA and replication study) and tumor site (colon, rectum and colorectal cancers). Sensitivity analysis was also performed to assess the influence of each individual study on overall estimates by sequential removal of individual studies [28]. Cumulative analysis was performed to investigate the dynamic trend of the association between the SNP and CRC with accumulation of studies by published year [29]. Publication bias was estimated by funnel plot and Eegger's test [30], [31]. All statistical analyses were carried out by Stata version 10.0.

## Results

### Results of case-control study

#### Population characteristics

A total of 641 incident cases of colorectal cancer and 1037 frequency-matched controls were enrolled in this study. As shown in Table 1, males were 59.9% among cases compared with 59.1% among controls. Mean age was56.31 years (±12.59) for cases and 57.24 years (±10.86) for controls. There was no significant difference in distribution of sex (*P* = 0.748) and age (*P* = 0.119) between case and control group. Of Cases, 39% had colon cancer and 61% had rectum cancer. Regarding tumor state, 12.9%, 35.6%, 35.4% and 16.1% of cases were classified as Duke's A, B, C and D stage at the time of diagnosis, respectively.

**Table 1:** Characteristics of study population.

Variables	Cases (N = 641)No. (%)	Control (N = 1037)No. (%)	*P*
Sex			0.784^a^
Male	384 (59.9)	613 (59.1)	
Female	257 (40.1)	424 (40.9)	
Age (years)	56.31±12.59	57.24±10.86	0.119^b^
Tumor site			
Colon	250 (39%)		
Rectum	391 (61%)		
Duke's stage			
A	83 (12.9%)		
B	228 (35.6%)		
C	227 (35.4%)		
D	103 (16.1%)		

a *P* value was calculated by the *x^2^* test;

b *P* value was calculated by the *t* test.

Genotypes in the controls conformed to Hardy-Weinberg equilibrium (*P* = 0.214). Significant difference in genotype distribution was observed between cases and controls (*χ^2^* = 21.25, *P*<0.001). In the multivariate logistic regression model, individuals with the CT genotype had a significant, 57% increased risk of CRC (OR = 1.57; 95% CI, 1.27–1.94, *P*<0.001) compared to those with the CC homozygote. Due to the low frequency of the TT genotype (3.2% in controls and 1.6% in cases) in this study population, a dominant model was perform, by combining the TT with the CT into an T carrier (TT plus CT) group, and result showed that the T carrier also present significantly increased risk, compared with those carrying the CC genotype (OR = 1.46; 95%CI, 1.19–1.80, *P*<0.001). In the allelic model, T allele carriers also showed significantly increased risk compared to those with the C allele (OR = 1.26; 95% CI, 1.05–1.51, *P* = 0.01). In the additive model, per-T allele similarly conferred an OR of 1.27 (95% CI, 1.05–1.52, *P* = 0.01) (Table 2).

**Table 2:** The association between rs4939827 and colorectal cancer risk in a Chinese population.

	Controls	Cases	OR (95% CI)^b^			
	CC/CT/TT	CC/CT/TT	CT vs. CC	TT vs. CC	Dominant model	Additive model
Total	732/272/33	399/232/10	1.57 (1.27–1.94)	–	1.46 (1.19–1.80)	1.27 (1.05–1.52)
Tumor site			–	–		
Colon	732/305^a^	149/101^a^	–	–	1.65 (1.24–2.20)	
Rectum	732/305^a^	250/141^a^	–	–	1.35 (1.06–1.73)	
Duke's stage			–	–		
A+B	732/305^a^	184/127^a^	–	–	1.67 (1.28–2.17)	
C+D	732/305^a^	215/115^a^	–	–	1.28 (0.98–1.67)	

a CC/(CT+TT);

b ORs and their corresponding 95% CIs were calculated by multivariate logistic regression model after adjusting for age and sex.

We then stratified data according to the pathological factors under the dominant model. The CT plus TT genotypes were both associated with increased risk of colon and rectal cancers. Interestingly, the effect of the CT plus TT genotypes was larger in colon cancer (OR = 1.65; 95%CI = 1.24–2.20) than that in rectum cancer (OR = 1.35; 95% CI = 1.06–1.73). Regarding the Duke's stage, the CT plus TT genotypes were associated with increased risk in early stage (A+B: OR = 1.67; 95% CI, 1.28–2.17) but not in advanced cancer (C+D: OR = 1.28; 95% CI, 0.98–1.67; Table 2).

### Results of meta-analysis

#### Study characteristics

A total of 11 publications plus the current study, comprising 25 case-control studies of 34313 cases and 33251 controls, were finally included in this meta-analysis[3], [10], [13]–[17], [32]–[35] , of which, 19 studies were conducted in European [3], [10], [16]–[17], [32]–[34], 5 in Asian [3], [13], [14], [35], and 1 in the mixed population [15] (Table 3). The report by Broderick et al. only provided data on allele frequency and thus was only included in the pooled analysis of allelic OR [10].

**Table 3:** Characteristics of studies on rs4939827 polymorphisms and risk of colorectal cancer included in the meta-analysis.

First author	Published year	Country	Ethnicity	Study method	Studydesign	Genotyping method	Tumor site	Case/control
Broderick P [10]	2007	UK	European	Nested CC	GWAS	Illumina	CRC	620/960
		UK	European	CC	Replication	Allele-PCR	CRC	4422/3844
		UK	European	CC	Replication	Allele-PCR	CRC	1992/1680
		UK	European	Nested CC	Replication	Allele-PCR	CRC	963/343
Tenesa A [3]	2008	Scotland	European	Nested CC	GWAS	Illumina	CRC	2895/3059
		Scotland	European	Nested CC	Replication	TaqMan-PCR	CRC	830/923
		Australia	European	CC	Replication	TaqMan-PCR	CRC	1318/1140
		Canada	European	CC	Replication	TaqMan-PCR	CRC	1173/1182
		England	European	CC	Replication	TaqMan-PCR	CRC	2232/2250
		German	European	CC	Replication	TaqMan-PCR	CRC	2150/2182
		Israel	European	CC	Replication	TaqMan-PCR	CRC	1352/1336
		Spain	European	CC	Replication	TaqMan-PCR	CRC	418/295
		Japan	Asians	CC	Replication	TaqMan-PCR	CRC	4391/3178
Curtin K [14]	2009	Leeds, UK	European	CC	Replication	SNPlex	CRC	245/216
		Sheffield, UK	European	CC	Replication	SNPlex	CRC	398/400
		Utah, USA	European	CC	Replication	SNPlex	CRC	422/425
Thompson CL [15]	2009	USA	Mixed	CC	Replication	TaqMan-PCR	Colon	554/709
Mates IN [16]	2010	Romania	European	CC	Replication	Centaurus	colon, rectum	92/95
Niittymaki I [32]	2010	Finland	European	CC	Replication	Sequencing	CRC	970/969
Slattery ML [33]	2010	USA	Americans	CC	Replication	TaqMan-PCR	Colon	1475/2287
Xiong F [17]	2010	China	Asians	CC	Replication	T-ARMS-PCR	Colon, rectum	2124/2180
von Holst [34]	2010	Sweden	European	Nested CC	Replication	deCode test	CRC	1782/1679
Li X [13]	2011	China	Asians	CC	Replication	Sequenom	CRC	138/168
Ho JW [35]	2011	HK, China	Asians	CC	Replicatoin	Sequenom	CRC	716/714
Zhu B	2011	China	Asians	CC	Replication	TaqMan-PCR	Colon, rectum	641/1037

Abbreviation: CC, case-control study; CRC, colorectal cancer.

#### Frequency of risk allele in control population

Both significant between-study heterogeneity were observed in European and Asian groups (*P* for heterogeneity <0.001). Under random-effects model, the pooled frequency of the T allele was 51.2% (95% CI, 50.1%–52.2%) in European controls, which was markedly higher than that of 23.4% in Asian controls (95% CI, 18.4%–28.3%)(Figure S2). These pooled frequencies were similar to those reported in HapMap database of 0.508 and 0.256 for European and Asian, respectively.

#### Overall meta-analysis of rs4939827 in associated with CRC

**Figure 1:**
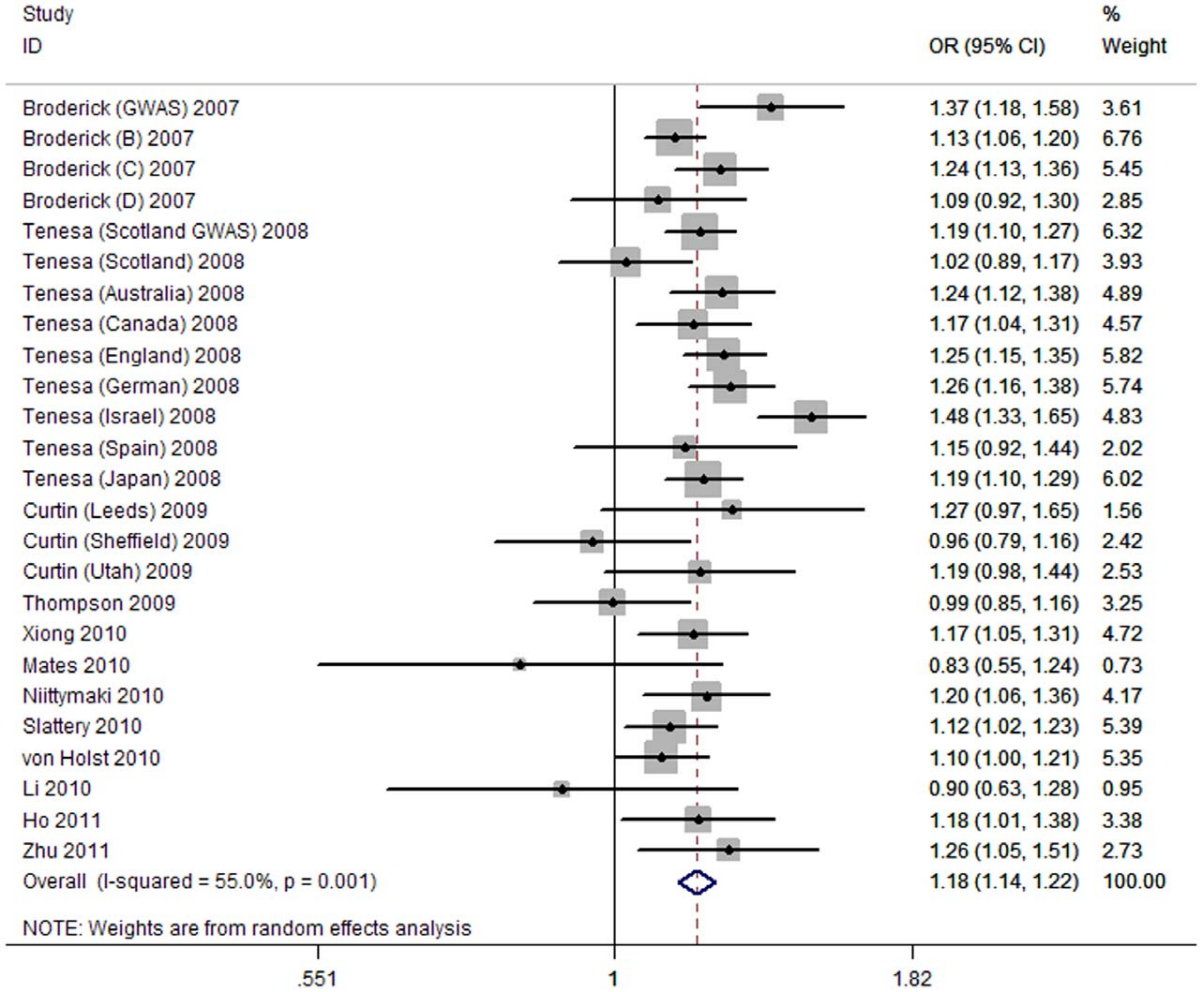
Forest plot of association of rs4939827 with CRC under allelic model.

As shown in Table 4, significant evidence of heterogeneity was seen in all genetic models (all *P* for heterogeneity <0.05), and ORs for all genetic models were pooled under random-effects model. In allelic model, the T allele conferring a pooled OR of 1.18 compared to the C allele (95% CI, 1.14–1.22; Figure 1). Genotypic ORs of the TT versus CC and CT versus CC were 1.33 (95%CI, 1.21–1.47) and 1.17 (95%CI, 1.09–1.26), respectively. Similarly, the dominant, recessive and additive models were all associated with significantly increased risk of CRC.

**Table 4:** Pooled OR with 95% CI for the association between rs4929827 and colorectal cancer risk in the meta-analysis.

	Genetic model	OR (95%CI)	*P*	*P* forHeterogeneity	*I^2^* (%)
Overall					
(n = 25)	T vs. C	1.18 (1.14–1.22)	<0.001	0.001	55.0
(n = 21)	TT vs. CC	1.33 (1.21–1.47)	<0.001	<0.001	60.6
	CT vs. CC	1.17 (1.09–1.26)	<0.001	<0.001	59.1
	Dominant	1.22 (1.14–1.31)	<0.001	<0.001	61.6
	Recessive	1.24 (1.17–1.32)	<0.001	0.039	38.4
	Additive	1.17 (1.14–1.20)	<0.001	0.001	54.8

#### Stratified analysis

To investigate the potential source of between-study heterogeneity, stratified analysis was performed (Table 5). After stratifying by ethnicity, significant heterogeneity still existed in European, whereas in Asian heterogeneity was effectively reduced. In European population, the variant in all genetic models presented significantly increased risk of CRC. In Asian population, all the genetic models except for the TT genotypic and recessive models were associated with increased risk of CRC, potentially suggesting that the T variant act in various manners between different ethnical populations. When stratified by sample size, we defined the large group when the sample size was more than 1000, otherwise was small group, heterogeneity was almost removed in small sample subgroup but not large sample subgroup. Significant association of CRC risk with the variant remained in large sample studies for all genetic models, whereas only the recessive model showed significant result in small sample subgroup. According to tumor site, only the data on dominant model was available. For colon cancer, heterogeneity was still observed and no significant association was found, whereas the dominant model was significantly associated with increased risk of rectum cancer without evidence of heterogeneity. Regarding study design, GWA studies were merely pooled in allelic model due to limited studies for assessing genotypic model. Heterogeneity did not change after stratifying by GWAS and replication and significant association still existed.

**Table 5:** Stratified analysis of the association between rs4939827 genotype and colorectal cancer risk.

Category	Genetic model	OR (95%CI)	*P*	*I^2^*	*P* forheterogeneity
**Ethnicity**					
European (n = 19)	T vs. C	1.18 (1.14–1.24)	<0.001	60.6	<0.001
European (n = 15)	TT vs. CC	1.39 (1.26–1.55)	<0.001	61.0	0.001
	CT vs.CC	1.17 (1.07–1.27)	0.001	58.6	0.002
	Dominant	1.24 (1.13–1.36)	<0.001	64.7	<0.001
	Recessive	1.26 (1.18–1.34)	<0.001	37.4	0.071
	Additive	1.18 (1.14–1.21)	<0.001	68.0	<0.001
Asians (n = 5)	T vs. C	1.18 (1.12–1.25)	<0.001	0.0	0.584
Asians (n = 5)	TT vs. CC	1.18 (0.93–1.51)	0.181	47.4	0.107
	CT vs.CC	1.23 (1.09–1.39)	0.001	54.4	0.067
	Dominant	1.24 (1.13–1.35)	<0.001	28.2	0.234
	Recessive	1.11 (0.86–1.43)	0.428	52.6	0.077
	Additive	1.18 (1.11–1.25)	<0.001	0.0	0.469
**Sample size**					
Large (n = 19)	T vs.C	1.19 (1.15–1.24)	<0.001	58.2	<0.001
Large (n = 15)	TT vs. CC	1.37 (1.24–1.52)	<0.001	66.0	<0.001
	CT vs.CC	1.22 (1.14–1.30)	<0.001	53.5	0.007
	Dominant	1.27 (1.18–1.35)	<0.001	59.3	0.002
	Recessive	1.24 (1.15–1.33)	<0.001	53.1	0.008
	Additive	1.18 (1.15–1.21)	<0.001	55.9	0.004
Small (n = 6)	T vs.C	1.07 (0.95–1.21)	0.244	26.8	0.234
Small (n = 6)	TT vs. CC	1.15 (0.91–1.45)	0.232	18.9	<0.001
	CT vs.CC	0.89 (0.69–1.13)	0.339	45.2	0.104
	Dominant	0.96 (0.76–1.22)	0.744	47.5	0.090
	Recessive	1.23 (1.05–1.43)	0.011	0.0	0.768
	Additive	1.05 (0.95–1.16)	<0.001	29.1	0.217
**Tumor site**					
Colon (n = 3)	T vs. C	1.07 (0.96–1.20)	0.201	35.7	0.212
Colon (n = 5)	Dominant	1.03 (0.77–1.38)	0.861	85.6	<0.001
Rectal (n = 3)	Dominant	1.24 (1.10–1.41)	0.001	0.0	0.725
Colorectal (n = 22)	T vs.C	1.19 (1.15–1.23)	<0.001	53.0	0.002
Colorectal (n = 16)	Dominant	1.20 (1.09–1.33)	<0.001	71.3	<0.001
**Design**					
GWAS (n = 2)	T vs.C	1.26 (1.09–1.44)	0.001	66.7	0.083
Replication (n = 23)	T vs. C	1.17 (1.13–1.22)	<0.001	55.5	0.001
Replication (n = 20)	TT vs. CC	1.32 (1.19–1.46)	<0.001	62.4	<0.001
	CT vs. CC	1.16 (1.07–1.25)	<0.001	59.2	<0.001
	Dominant	1.21 (1.12–1.30)	<0.001	62.1	<0.001
	Recessive	1.24 (1.16–1.33)	<0.001	39.3	0.037
	Additive	1.17 (1.14–1.20)	<0.001	56.9	0.001

#### Sensitivity analyses and cumulative meta-analysis

Due to the significant between-study heterogeneity for all genetic models, sensitivity analysis was performed, by removing the individual studies sequentially under random-effects model, to assess the effect of each study on the pooled estimate. As shown in Table 6, the pooled OR for the allelic model was similar before and after elimination of each study. Similar results were seen for other genetic models that no single study dramatically change the pooled ORs, indicating the robust stability of the current results.

**Table 6:** Sensitivity analysis of allelic model.

Study omitted	OR (95%CI)	*P* for heterogeneity	*I^2^*
Broderick 2007 (GWAS) [10]	1.17 (1.13–1.22)	0.001	53.5%
Broderick 2007 (B)	1.18 (1.14–1.23)	0.001	54.6%
Broderick 2007 (C)	1.17 (1.13–1.22)	<0.001	56.1%
Broderick 2007 (D)	1.18 (1.14–1.23)	<0.001	56.2%
Tenesa 2008 (Scotland GWAS) [3]	1.17 (1.13–1.22)	<0.001	56.9%
Tenesa 2008 (Scotland Replication)	1.19 (1.14–1.23)	0.001	53.7%
Tenesa 2008 (Australia)	1.18 (1.13–1.22)	<0.001	56.3%
Tenesa 2008 (Canada)	1.18 (1.13–1.22)	<0.001	56.9%
Tenesa 2008 (England)	1.17 (1.13–1.22)	0.001	55.6%
Tenesa 2008 (German)	1.17 (1.13–1.22)	0.001	54.9%
Tenesa 2008 (Israel)	1.17 (1.13–1.20)	0.046	35.3%
Tenesa 2008 (Spain)	1.18 (1.14–1.22)	<0.001	56.9%
Tenesa 2008 (Japan)	1.18 (1.13–1.22)	<0.001	56.9%
Curtin 2009 (UK-Leeds) [17]	1.18 (1.13–1.22)	<0.001	56.7%
Curtin 2009 (UK-Sheffield)	1.18 (1.14–1.23)	0.001	53.9%
Curtin 2009 (USA-Utah)	1.18 (1.13–1.22)	<0.001	56.9%
Thompson 2009 [15]	1.19 (1.14–1.23)	0.001	52.6%
Xiong 2010 [13]	1.18 (1.13–1.22)	<0.001	56.9%
Mates 2010 [16]	1.18 (1.14–1.22)	0.001	54.3%
Niittymaki 2010 [32]	1.18 (1.13–1.22)	<0.001	56.9%
Slattery 2010 [33]	1.18 (1.14–1.22)	0.001	55.6%
von Holst 2010 [34]	1.18 (1.14–1.23)	0.001	54.7%
Li 2011 [14]	1.18 (1.14–1.23)	0.001	54.9%
Ho 2011 [35]	1.18 (1.13–1.22)	<0.001	56.9%
Zhu 2011	1.18 (1.13–1.22)	<0.001	56.6%
Combined	1.18 (1.14–1.22)	0.001	55.0%

**Figure 2:**
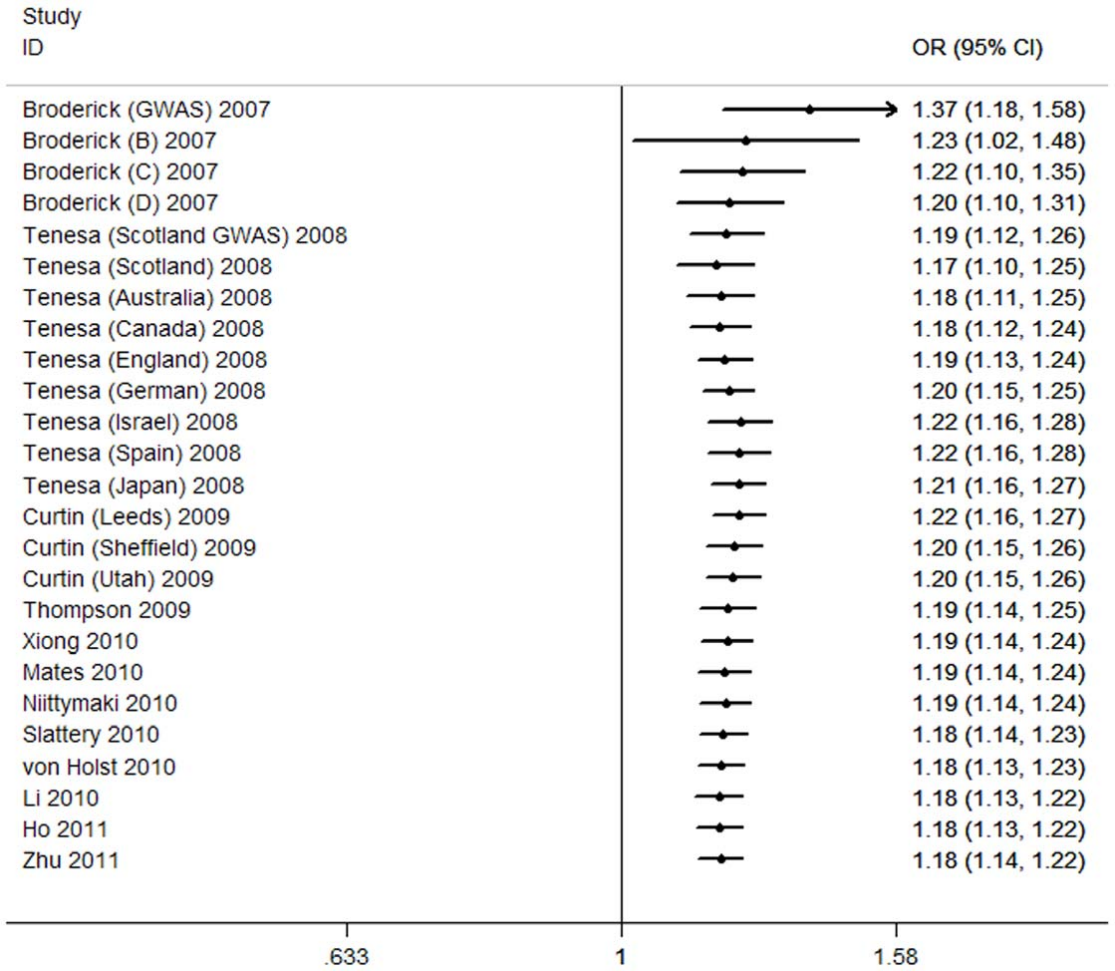
Cumulative meta-analysis of association rs4939827 with CRC under allelic model.

Accumulative meta-analysis was carried out via the assortment of studies by publication time. As shown by Figure 2, in the allelic model, the 95% CIs for the pooled OR became increasingly narrower with each accumulation of more studies, indicating the progressively boosted precision of the estimation by continual adding more samples. Simultaneously, inclinations toward significant association were evident over time. Similar results were seen in other genetic models.

#### Publication Bias

As reflected by the funnel plot (Figure S3.) and Egger's test, no evidence of publication bias was observed in all genetic models (all *P* for egger's test>0.05).

## Discussion

rs4939827 located at 18q21 was revealed to be associated with CRC risk by two GWA studies, but inconsistent results have been reported by multiple following replication studies. In this study, we initially found a significant association between the variant 4939827 and increased risk of CRC in a case-control set of a Chinese population. Then the following meta-analysis, first to integrate GWA and replication data from 25 case-control studies of 34313 cases and 33251 controls, consistently indicated the significant association of rs4939827 with the risk of CRC. This significant association was further confirmed by cumulative meta-analysis, presenting the effect of the variant got increasingly significant with each accumulating of more data over time. rs4939827 is located in intron 3 of *SMAD7*,which encodes a inhibitory SMAD protein that function as a negatively feedback regulator of TGF-β signals [36]. There was evidence that the over-expression of SMAD7 could promote tumorigenesis via disturbing TGF-β-induced growth inhibition and apoptosis. Although we herein confirmed the association between the rs4939827 and CRC risk, whether this SNP is causative was still uncertain. Intriguingly, Houlston et al. have identified a novel C to G SNP unlisted in dbSNP (MAF = 0.47), through re-sequencing the linkage disequilibrium (LD) region tagged by rs4939827 in 2532 CRC cases and 2607 controls, was maximally associated with CRC risk [37]. The following functional models further provided evidence for the role of this SNP in transcription factor binding, proposing that this functional SNP was likely to be one of the causal variants in susceptibility loci tagged by rs4939827.

Nevertheless, the obvious evidence of between-study heterogeneity in this meta-analysis should be issued. We have applied a comprehensive stratified analysis to interrogate the potential source of heterogeneity. After stratifying by ethnicity, heterogeneity was largely reduced in Asian, reflecting ethnicity could partly explain the heterogeneity. The further supports of that came from the evidence that various manners the T variant likely act in and different allele frequencies between European and Asian populations. When stratified by tumor sites, rectum cancer subgroup did not show heterogeneity anymore, suggesting tumor sites might also be a potential source of heterogeneity. Additionally, significant association only presented for rectum cancer, which was inconsistent with our case-control study, possibly due to our small sample size for stratification by tumor sites. Regarding sample size, heterogeneity was almost removed in small sample studies but not large sample subgroup, possibly due to more complex confounding factors introduced into large sample. Significant association remained in large sample studies for all genetic models, whereas only the recessive model showed significant result in small sample subgroup, reflecting the limited power of small sample size to detect the modest effect of the variant. Taken together, we revealed that the ethnicity, tumor sites, and sample size might constitute source of heterogeneity in this meta-analysis. Whilst, the significant association of rs4939827 presented in the subgroup of replication studies, consistent with the result from GWA study subgroup, suggested this meta-analysis succeed in amplifying power to detect the modest effect of this variant by pooling data across studies. Furthermore, the sensitivity analysis and publication bias assessment indicated the current results from this meta-analysis were stable.

Despite the clear strengthen of this study that applied a comprehensive analysis strategy, several limitations should be Figured out. First, the sample size of our case-control study was relatively small. Nevertheless, the following meta-analysis with enough power has drawn the consistent result with our case-control study. Second, the analysis of separate effect for colon or rectum cancer conferred limited power, and more studies are needed. Additionally, CRC is a complex trait corporately influenced by genetic and environmental factors; however, lacking the environment data limited us to further assess gene-environment interaction.

In conclusion, our study in a Chinese population and this meta-analysis collectively confirm the significant association between SNP rs4939827 and the colorectal cancer risk in European and Asian populations. However, although a novel SNP in highly LD with rs4939827 has been proposed to be causal, further fine-mapping of the CRC susceptibility loci tagged by rs4939827 is warranted to uncover more causal variants, especially for the low-frequency or rare functional variants.

## Supporting Information

Figure S1 **Flow chart of study selection.**
(TIF)

Figure S2 **Pooled frequency of T allele in European and Asian population.**
(TIF)

Figure S3 **Funnel plot of publication bias under allelic model.**
(TIF)
