# Supplementary figures and images for: A Common SMAD7 Variant Is Associated with Risk of Colorectal Cancer: Evidence from a Case-Control Study and a Meta-Analysis
Source: PLoS One. 2012 Mar 21;7(3):e33318. doi: 10.1371/journal.pone.0033318 (PMC3310071; doi:10.1371/journal.pone.0033318)

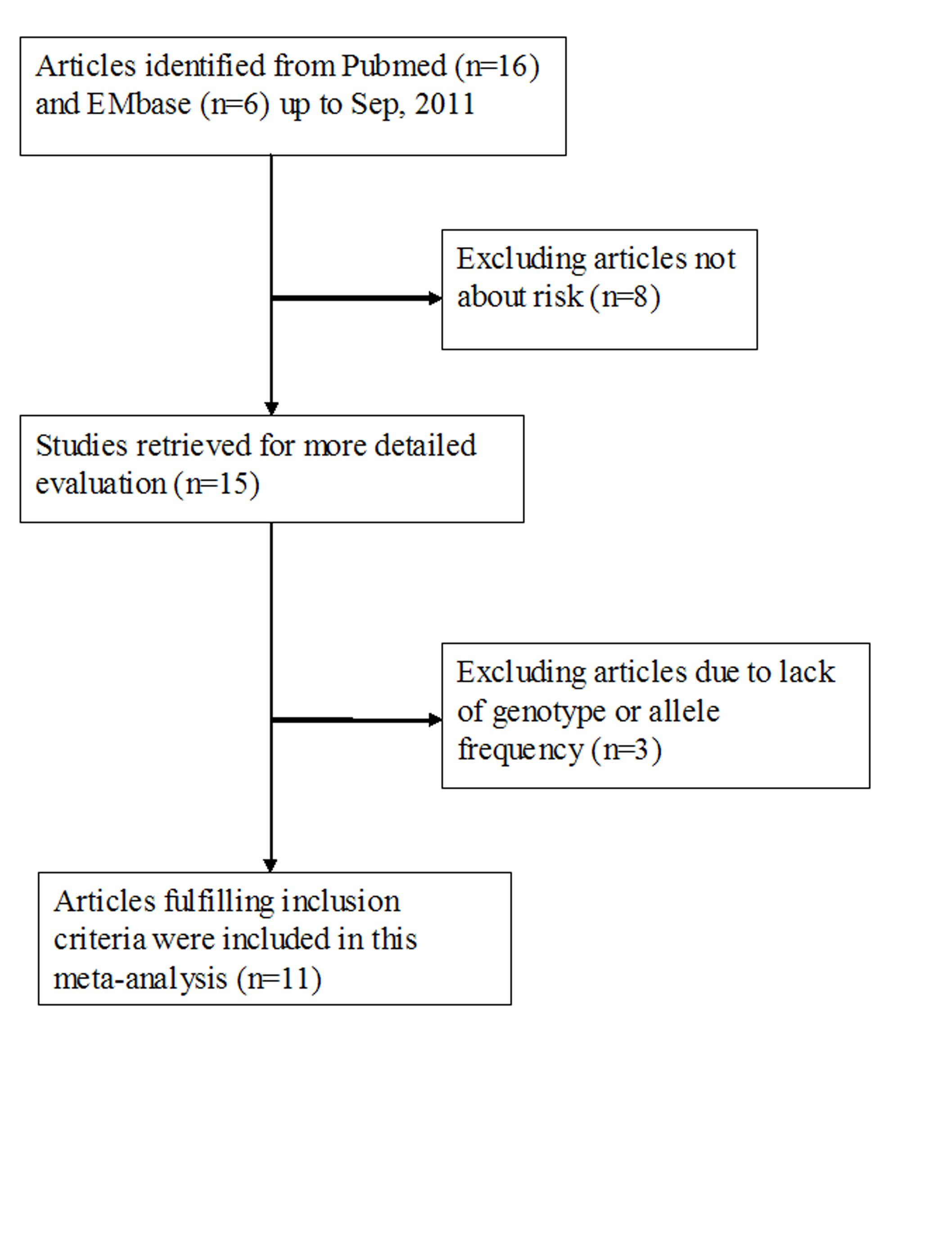

Supplement: Figure S1 — Flow chart of study selection. (TIF) [file pone.0033318.s001.tif]

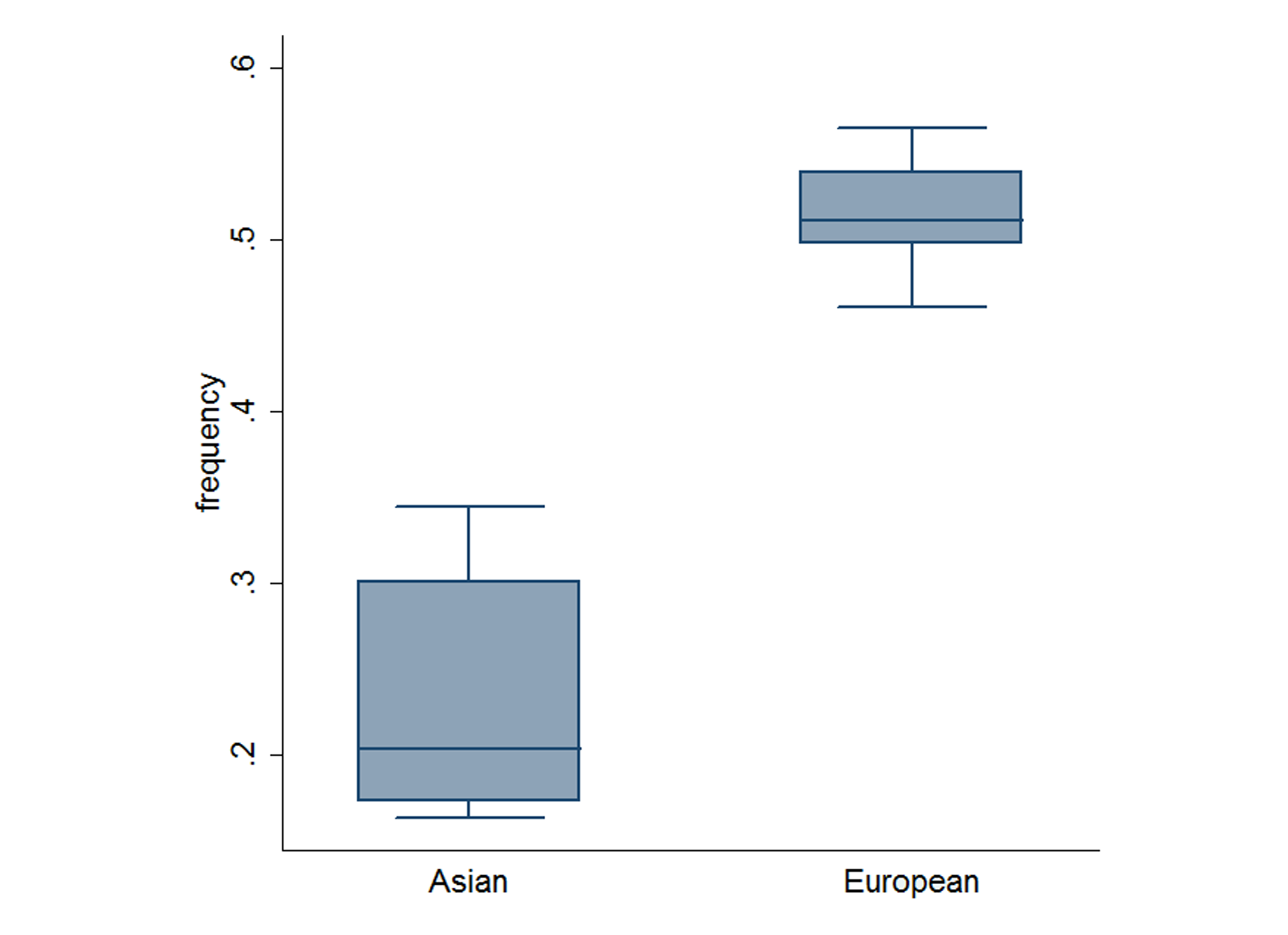

Supplement: Figure S2 — Pooled frequency of T allele in European and Asian population. (TIF) [file pone.0033318.s002.tif]

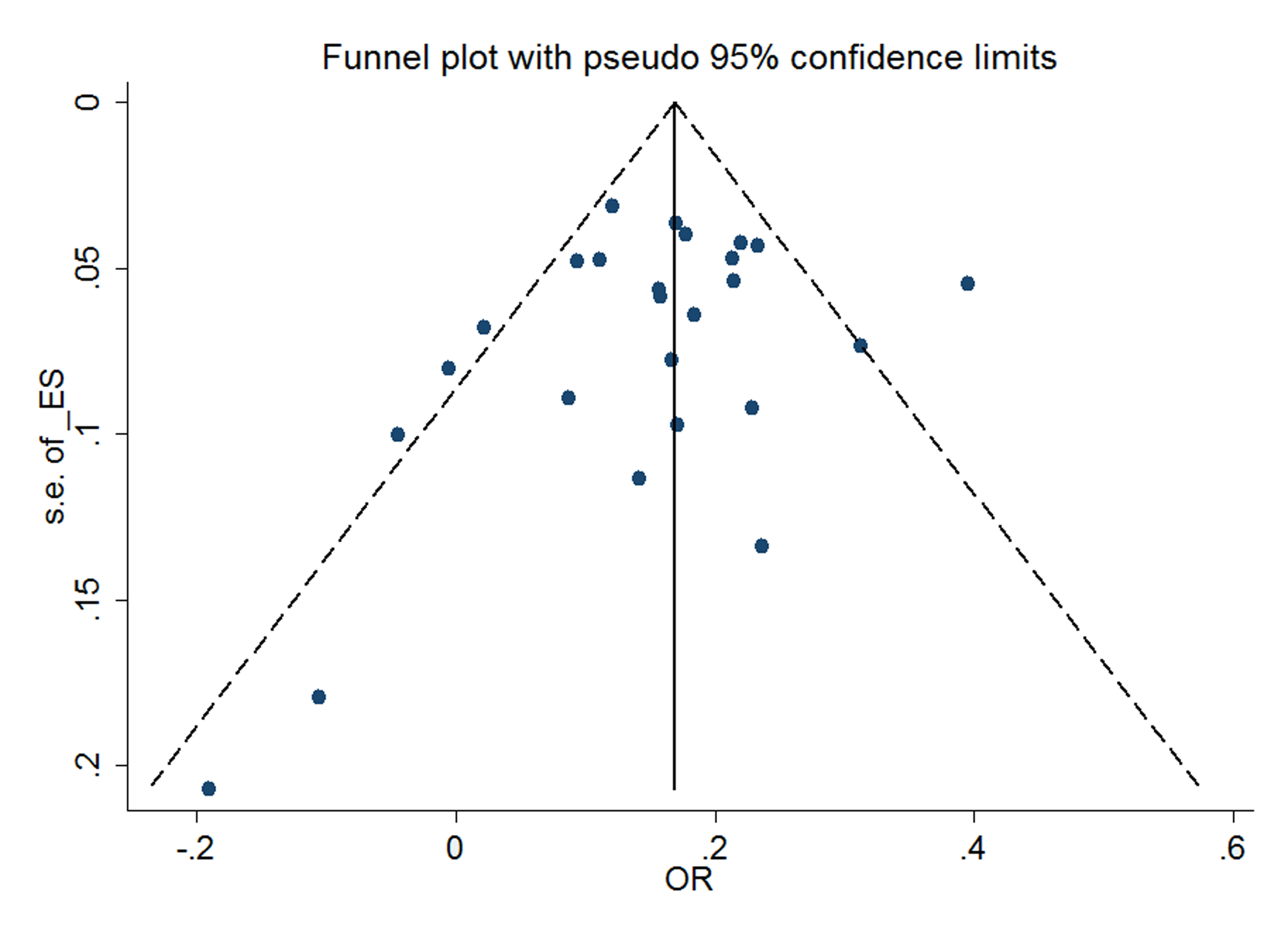

Supplement: Figure S3 — Funnel plot of publication bias under allelic model. (TIF) [file pone.0033318.s003.tif]
